# Retreatability of Root Canals Obturated Using Gutta-Percha with Bioceramic, MTA and Resin-Based Sealers

**Published:** 2015-03-18

**Authors:** Emel Uzunoglu, Zeliha Yilmaz, Derya Deniz Sungur, Emre Altundasar

**Affiliations:** a *Hacettepe University, Dental School, Department of Endodontics, Ankara, Turkey*

**Keywords:** Bioceramics, Epoxy Resin, Mineral Trioxide Aggregate, Retreatment, Root Canal Sealer

## Abstract

**Introduction:** The aim of this study was to evaluate the retreatability of root canals obturated with gutta-percha (GP) and three different endodontic sealers [iRoot SP (bioceramic sealer), MTA Fillapex (MTA-based sealer) and AH-26 (epoxy resin-based sealer)] using the ProTaper Universal Retreatment (PTR) system. **Methods and Materials:** Forty extracted single-rooted human teeth were prepared with universal ProTaper files up to F4 (40/0.06). Specimens were randomly divided into four groups according to obturation technique/material: single-cone GP/AH-26, lateral compaction of GP/AH-26, single-cone GP/iRoot SP, and single-cone GP/MTA Fillapex. Root fillings were removed with PTR. The time taken to reach the working-length (TWL) was recorded. Roots were longitudinally sectioned and each half was evaluated using a stereomicroscope. Three observers scored each third of all specimen. Obtained data were analyzed using the Kruskal-Wallis, Mann-Whitney U, Welch and Games-Howell tests. The level of significance was set at 0.05. **Results:** In single-cone GP/MTA Fillapex group the TWL was significantly shorter. The remnant of filling material in the apical and middle thirds of groups was similar and higher than the coronal thirds. **Conclusion:** None of the tested sealers were completely removed from the root canal system.

## Introduction

Although endodontic treatment has a high and predictable success rate, failures may still occur and post-endodontic disease can develop [1]. The failure rate of root canal treatments has been reported to be 14 to 16% [2]. In cases of treatment failure, non-surgical retreatment, surgical procedures or tooth extraction may be chosen [3]. 

The success of orthograde retreatment depends on adequate cleaning and shaping of the previously untouched areas of the root canal system. Therefore, special attention should be paid to the complete removal of root filling material [4, 5]. Apart from the retreatment modality, the filling technique, type of used filling material and sealer can affect the removability of the root filling [6]. 

Root canal sealers are used to obturate the canal irregularities and fill the voids between root canal filling and canal walls [7-9]. Sealers are based on zinc oxide-eugenol, calcium hydroxide, glass-ionomer, silicone, polymer resins [7, 8, 10] and calcium silicate [9, 11]. Epoxy resin sealers have high bond strength to dentin [12, 13] and it is reported that they leave higher amounts of root filling remnants after retreatment [14]. 

Calcium silicate-based sealers have been proposed as endodontic filling materials because of their excellent biocompatibility, bioactivity, and osteoconductivity [11, 15, 16]. iRoot SP (Innovative BioCeramix Inc, Vancouver, BC, Canada) is a bioceramic-based sealer composed of biocompatible nanosphere components such as tricalcium silicate, dicalcium silicate, calcium phosphate monobasic, amorphous silicon dioxide and tantalum pentoxide [17, 18]. It has excellent physical and antimicrobial properties and can be used for filling the root canals with or without GP [12, 16, 19]. The push-out bond strength and retreatability of iRoot SP is reported to be similar to that of AH-Plus [19, 20]. The sealer sets in contact with dentinal moisture [16].

Aiming at achieving the biological and sealing properties of mineral trioxide aggregate (MTA), sealers with the basis of MTA have been introduced. MTA Fillapex (Angelus, Londrina, PR, Brazil) is a radiopaque, insoluble sealer that apart from MTA, is composed of resins, radiopaque bismuth, nano-particulated silica and pigments. The required setting hydration is taken from surrounding dentin [11].

One of the basic properties of an ideal root canal filling material is being removable for retreatment purposes [21]. For proper removal of root canal filling, many techniques and materials have been proposed including hand files, heat-carrying instruments, chemical solvents, ultrasonic devices, lasers and engine-driven instruments such as Gates Glidden drills, NiTi rotary instruments and rotary instruments [14, 22-25]. Specific rotary retreatment kits were introduced to facilitate this challenging procedure. The ProTaper Universal Retreatment (PTR) system (Dentsply Maillefer, Ballaigues, Switzerland) includes three instruments with various tapers and diameters at the tip (D1 30/0.09, D2 25/0.08 and D3 20/0.07). D1 has a cutting tip to facilitate initial penetration into filling material. D2 and D3 both have non-cutting tips and are used to remove the obturating material from the mid and apical thirds, respectively [26]. 

The aim of the present study was to evaluate the retreatability of root canals obturated with gutta-percha (GP) and three different sealers including iRoot SP (bioceramic sealer), MTA Fillapex (MTA-based sealer) and AH-26 (epoxy resin-based sealer), using PTR.

## Materials and Methods

A total of forty extracted straight-rooted mature human mandibular premolars with single canals (verified radiographically) were disinfected in 1% sodium hypochlorite (NaOCl) and then stored in a 0.1% thymol solution. The teeth were examined under 25× magnification of an operating microscope (Zeiss, Oberkochen, Germany) and those with microcracks were excluded from the study. 

The crowns of teeth were removed with a water-cooled, double-faced diamond disc to form standardized root samples with 15 mm lengths. A #10 K-file (Dentsply Maillefer, Ballaigues, Switzerland) was inserted in the canal until it was visible at the apical foramen and the working length (WL) was determined by subtracting 1 mm from this measurement. The root canals were prepared using ProTaper Universal Rotary System (Dentsply Maillefer, Ballaigues, Switzerland) to size F4 (40/0.06), according to the manufacturer’s instructions. Instruments were discarded after preparing five canals. Irrigation with 2 mL of a 5.25% NaOCl solution was performed during filing. Finally, to remove the smear layer, 17% ethylenediaminetetraacetic acid (EDTA) was applied for 1 min followed by 2 mL of 5.25% NaOCl. Then the canals were flushed with saline and dried.

Samples were randomly divided into four groups (*n*=10) based on root filling procedure: *1-*single-cone GP (#F4, 40/0.06, Dentsply Maillefer, Ballaigues, Switzerland) and AH-26 sealer (Dentsply, De Trey, Konstanz, Germany), *2-*lateral compaction of GP (MAF#40) and AH-26 sealer, *3-*single-cone GP and iRoot SP (Innovative BioCeramix Inc, Vancouver, BC, Canada) and *4-*single-cone GP and MTA Fillapex (Angelus, Londrina, PR, Brazil). Except for iRoot SP that is provided in ready to use syringes by the producer, two other sealers were prepared according to the manufacturers’ instructions. In all samples the root canal walls were dried with paper points (#25, Dentsply Maillefer, Ballaigues, Switzerland) and then the GP cone was coated with sealer and inserted into the root canal. The access cavities were temporarily sealed (Cavit-G, ESPE-Premier, Norristown, PA, USA) and the teeth were then stored in a humidified chamber (100% humidity and 37^°^C) for 2 weeks to allow the sealers to set. 

The root fillings were removed with PTR system (Dentsply Maillefer, Ballaigues, Switzerland) following the manufacturer’s instructions. The D1, D2, and D3 instruments were sequentially used in a crown-down manner with a brushing action to reach the WL until no more debris could be seen on the last file (D3) [24, 27]. The time required to reach the WL (TWL) was recorded with a chronometer in sec excluding the time for instrument changes and irrigation. No solvent was used to soften the GP. Finally, a #40 hand file was inserted into the root canal until it reached the WL without resistance. The canals were irrigated with a 5.25% NaOCl between files. To reduce inter-operator variability, a single operator carried out all root canal instrumentation and the retreatment procedure [28, 29]. 

Teeth were then grooved buccolingually with a diamond disc and then sectioned longitudinally. All root halves were evaluated under a stereomicroscope (Olympus Corporation, Taiwan) with 5× magnification and photographs were taken. Evaluation of GP remnants was done by direct visual scoring of the images obtained via a stereomicroscope. Three blinded operators performed the evaluation together and reached an agreement on the final score. A grading system, was used to score the amount of filling material residues at the coronal, middle, and apical portions of each canal as follows: *score 1*-no or slight presence (0%-25%) of debris on dentin surface; *score 2*-presence of some debris (25-50%) on dentin surfaces; *score 3*-presence of moderate amounts debris (50-75%) on dentin surfaces and *score 4*-heavy presence (>75%) of debris on dentin surface[26, 27].

The data was analyzed using the SPSS software (Version 13, SPSS, Chicago, IL, USA). The cleanliness of the root canal walls was analyzed using the Kruskal–Wallis and Mann-Whitney U tests with the Bonferroni correction and the TWL was analyzed by Welch ANOVA and Games-Howell tests at a significance level of 0.05. Intergroup comparison of data from each third of the canals was performed and then intragroup comparison was done.

**Figure 1:**
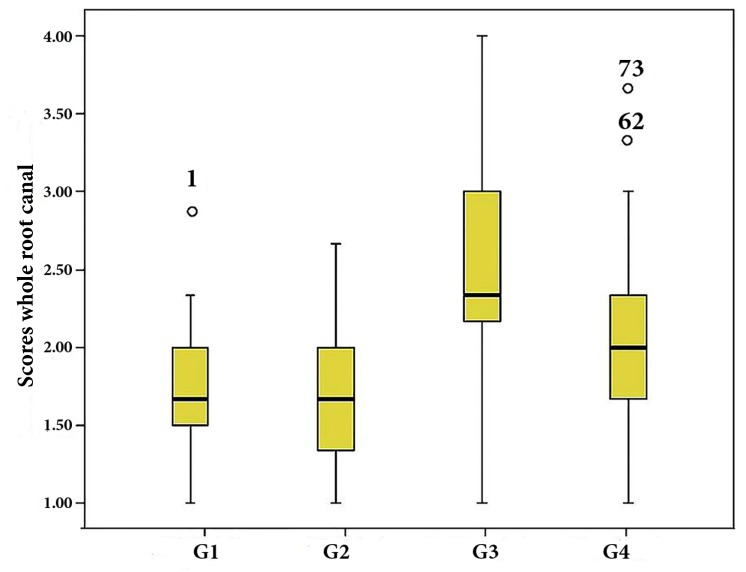
Plots of the scores given to the amount of remaining filling material within the whole canal lengths

## Results

Complete removal of the filling material from the root canal walls was not achieved in any of the groups. When the total canal length was observed, single-cone GP/iRoot SP presented significantly more remaining filling material than single-cone GP/AH-26 and lateral compaction of GP/AH-26 (*P*<0.001 for both comparisons) while there was no statistically significant difference between single-cone GP/iRoot SP and single-cone GP/MTA Fillapex (*P*=0.068) (Figure 1). 

**Figure 2:**
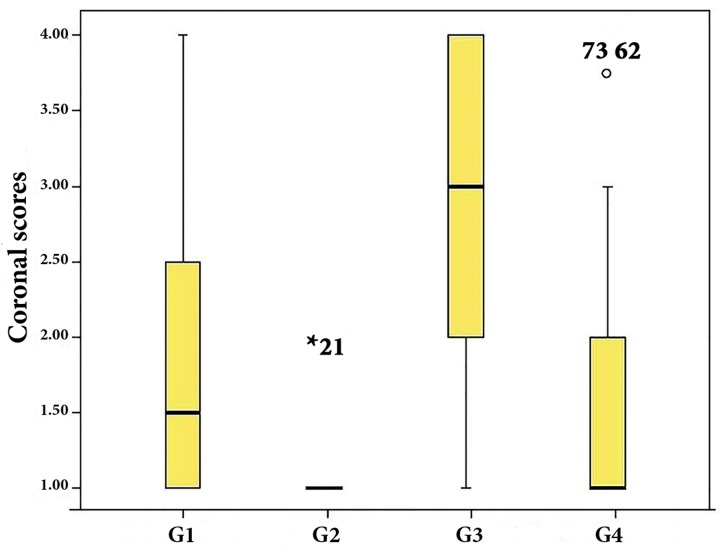
Plots of the scores dedicated to remaining filling material in the coronal third of all groups

There were no significant differences between the apical and middle thirds of groups considering the remaining filling material (*P*=0.187 and 0.163, respectively). However, there was more remaining filling material in the coronal third of the canals in single-cone GP/iRoot SP group compared to lateral compaction of GP/AH-26 and single-cone GP/MTA Fillapex (*P*<0.001, *P*=0.006, respectively) (Figure 2). Intergroup analysis revealed that in lateral compaction of GP/AH-26, there was significantly more filling material remaining in the coronal third than the middle and apical thirds (*P*=0.006 and 0.001, respectively), while there was no significant difference among different thirds of other groups (*P*=0.23 for single-cone GP/AH-26, *P*=0.19 for single-cone GP/iRoot SP and *P*=0.76 for single-cone GP/MTA Fillapex). 

**Figure 3:**
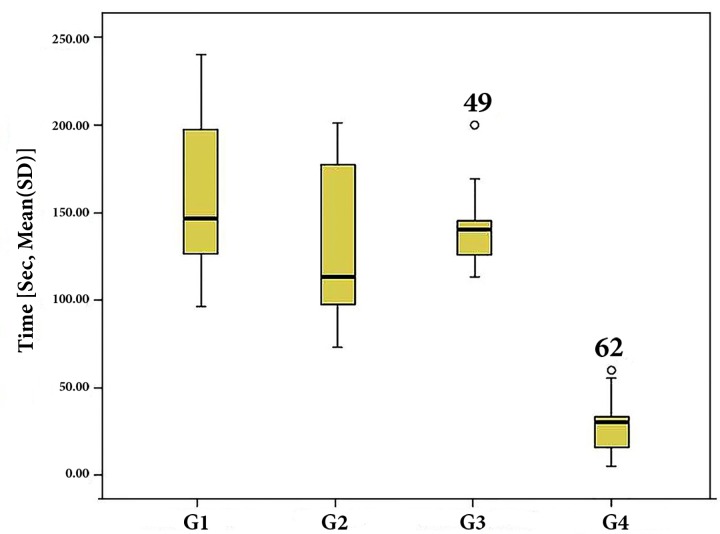
Plots of the time (sec) taken to reach the working length (TWL) for each group

The TWL in single-cone GP/MTA Fillapex was significantly shorter than the other groups (*P*<0.001) (Figure 3) and there were no statistically significant differences between other groups in this regard (*P*>0.05).

## Discussion

The present study evaluated the retreatability of root canals obturated with GP and iRoot SP, MTA Fillapex and AH-26 root canal sealers. The results revealed that complete removal of the filling material from the root canal was not achieved in any of the groups. 

New root canal filling materials have been introduced to increase the success of endodontic treatment; however, to fulfill the criteria of ideal materials for this purpose, they must be easily removable when retreatment is needed [21]. Removing root canal filling materials, including GP and sealer, from obturated root canals is essential for uncovering the remnants of necrotic tissues or bacteria that may be responsible for the persistent post treatment disease [20, 21]. The complete removal of filling material provides a corono-apical path and enables bacterial reduction through chemical/mechanical disinfection of the root canal system and dentinal tubules [20].

Micro-computed tomography (µ-CT), cone-beam computed tomography (CBCT), radiography, tooth splitting and direct visualization by stereomicroscopes or digital cameras and making the teeth transparent are common ways of assessing the remaining filling materials in the root canal system [14, 26, 28-30]. In this study, direct visual scoring of the images obtained via a stereomicroscope was performed for the evaluation of residual GP and sealer on the canal walls after longitudinal splitting of the samples. Direct visual scoring has been considered as a simple and efficient method [27]. However, displacement of the filling debris may have occurred during splitting that could possibly affect the accuracy of scoring. 

Removal of GP with hand files with/without solvents is time consuming, particularly when filling materials are highly condensed [31]. The use of NiTi rotary instruments has been recommended for GP removal and various studies have reported their efficacy, cleaning ability and safety. Furthermore, the use of NiTi rotary instruments during retreatment may decrease patient and operator’s fatigue [28]. In the present study, no solvent was used in conjunction with the rotary instruments. Wilcox *et al.* [32] showed that the use of solvents results in deposition of a thin layer of filling material on the root canal walls that is difficult to detect and remove. In addition, Gu *et al.* [29] evaluated the efficiency of PTR and showed that the cleanliness of canal walls was lower in groups retreated with a solvent. However, Madani *et al.* [26] evaluated the efficacy of D-RaCe, PTR and hand H-files in removal of gutta-percha and AH-plus with the aid of chloroform and reported similar efficacy of rotary and hand files. To minimize the number of variables involved in this study, extirpation of GP was done without using a solvent.

When there is no observable filling material left on the instruments, retreatment can be considered complete [5, 24, 27, 31]. However, in the present study despite ensuring the absence of visible obturating material on the instruments, all canals revealed filling material remnants during visual observation. Thus, it is evident that a lack of filling material on the instruments is not a valid criterion to demonstrate complete removal of filling material from the root canal walls. In this study, none of the filling materials could be completely removed from the root canal walls, similar to results reported in other studies [5, 26-28]. Previous studies have reported that the majority of the filling remnants on the canal walls were sealer based [27, 31]. Properties of the sealers such as adhesion to dentine and GP, penetration into the dentinal tubules, film thickness, dimensional changes and solubility may affect their removability [5, 33, 34]. In the present study, apical, middle, and coronal scorings among the groups were similar, with the exception that the coronal part of iRoot SP included more debris than the others. 

For the NiTi instruments, root filling removal in the coronal third is facilitated by the dental anatomy in this region and speed of their rotation [35]. Moreover, the temperature increase due to the rotating automated systems, results in plasticization of GP and facilitates its removal from the coronal third, which is the critical area with the highest concentration of filling material [34, 35]. As demonstrated in a previous study, a greater amount of filling material remained in the apical third than in the middle and coronal thirds, irrespective of the sealer used [14, 20]. One reason for this result is that anatomical variations are greater in the apical third [36]; another reason is the differences between tip sizes and tapers of F4 (40/0.06), and D3 (20/0.07) instruments used for canal retreatment and primary preparation, respectively. 

There were statistically significant differences regarding the amount of remaining filling material after retreatment of roots filled with AH-26, iRoot SP and MTA Fillapex, regardless of the obturation technique. The greater amount of remaining root filling material after retreatment was observed with iRoot SP and MTA Fillapex sealers. However, this result is contrary to the findings of Neelakantan *et al.* [37], who noted that the MTA-based sealer showed fewer remnants than the epoxy resin-based sealer (AH-Plus). This may be attributed to following factors: different evaluation methods between the two studies (scoring in the present study versus CBCT) and storage time for sealer setting (two weeks in the present study versus two months) and also adhesion properties of root canal sealers [12, 19]. Nagas *et al.* [19] showed that iRoot SP has higher dentine bond strength than AH-plus and MTA Fillapex. On the other hand, Forough Reyhani *et al.* [11] reported that the resin-based sealer (Epiphany) has higher bond strength to dentine than MTA Fillapex.

The TWL in the group filled with MTA Fillapex was significantly less than other groups. In another study it has also been reported that the retreatment time for MTA Fillapex was significantly shorter than that of the epoxy resin-based sealer (AH-Plus) [37]. The shorter retreatment time for MTA Fillapex can be related to its lower dentin bond strength [11, 12, 19]. The TWL in the groups filled with either lateral condensation or single-cone GP/AH-26 or iRoot SP was similar. This result can be correlated with similar obturation quality and similar push-out test results [38, 39]. 

It is interesting to note that there was more remaining debris in MTA Fillapex group with significantly less TWL. All sealers displayed different levels of viscosity, which resulted in different hardness of set materials. This difference in hardness may have affected the removal time of the filling material.

Comparison of canal cleanliness obturated with sealers from three different basis (AH-26, iRoot SP and MTA Fillapex) showed more filling materials remaining in the coronal third than in the apical and middle thirds. However, removal of MTA Fillapex was faster than that of the other test materials although more remnants were revealed with this sealer.

## Conclusion

None of the sealers could be completely removed from root canal walls. Moreover the extent of remnant filling material was independent of the time required to remove the filling materials.
